# The biology of ergothioneine, an antioxidant nutraceutical

**DOI:** 10.1017/S0954422419000301

**Published:** 2020-12

**Authors:** Irina Borodina, Louise C. Kenny, Cathal M. McCarthy, Kalaivani Paramasivan, Etheresia Pretorius, Timothy J. Roberts, Steven A. van der Hoek, Douglas B. Kell

**Affiliations:** 1 The Novo Nordisk Foundation Center for Biosustainability, Building 220, Chemitorvet 200, Technical University of Denmark, 2800 Kongens Lyngby, Denmark; 2 Department of Women’s and Children’s Health, Institute of Translational Medicine, University of Liverpool, Crown Street, Liverpool L8 7SS, UK; 3 Irish Centre for Fetal and Neonatal Translational Research (INFANT), Cork University Maternity Hospital, Cork, Republic of Ireland; 4 Department of Pharmacology and Therapeutics, Western Gateway Building, University College Cork, Cork, Republic of Ireland; 5 Department of Physiological Sciences, Faculty of Science, Stellenbosch University, Stellenbosch, Private Bag X1 Matieland, 7602, South Africa; 6 Department of Biochemistry, Institute of Integrative Biology, Faculty of Health and Life Sciences, University of Liverpool, Crown Street, Liverpool L69 7ZB, UK

**Keywords:** Ergothioneine, SLC22A4, Oxidative stress, Cytoprotectants, Nutraceuticals

## Abstract

Ergothioneine (ERG) is an unusual thio-histidine betaine amino acid that has potent antioxidant activities. It is synthesised by a variety of microbes, especially fungi (including in mushroom fruiting bodies) and actinobacteria, but is not synthesised by plants and animals who acquire it via the soil and their diet, respectively. Animals have evolved a highly selective transporter for it, known as solute carrier family 22, member 4 (SLC22A4) in humans, signifying its importance, and ERG may even have the status of a vitamin. ERG accumulates differentially in various tissues, according to their expression of SLC22A4, favouring those such as erythrocytes that may be subject to oxidative stress. Mushroom or ERG consumption seems to provide significant prevention against oxidative stress in a large variety of systems. ERG seems to have strong cytoprotective status, and its concentration is lowered in a number of chronic inflammatory diseases. It has been passed as safe by regulatory agencies, and may have value as a nutraceutical and antioxidant more generally.

## Introduction

Most of the classical vitamins such as vitamins A, B_1_, B_2_, B_3_, C, D, etc., were discovered by means of the fact that an inadequacy in their supply led to overt forms of deficiency disease such as blindness, beri-beri, pellagra, scurvy, rickets and so on. Consequently, it was easy to establish those food sources that contained such vitamins, since they relieved or prevented the relevant syndromes^(1,2)^. It is correspondingly hard, by these means, to detect the presence of a vitamin if it is present in virtually every foodstuff that an individual consumes. Recently, however, l-(+)-ergothioneine, hereafter ergothioneine (ERG), has emerged^(3–10)^ as an important nutrient, and indeed possible vitamin^(3)^, that has precisely these properties of a very widespread occurrence coupled, commonly, to a functional undersupply.

A related class of nutrient, which has not been demonstrated as necessary or essential for life yet provides health benefits when added at levels greater than a normal diet generally provides, has come to be known as nutraceuticals, a coinage based on an amalgamation of ‘nutrition’ and ‘pharmaceutical’^(11)^. Interest in such nutraceuticals, also known as ‘functional foods’, has increased enormously over the last few decades^(11–22)^ as our understanding of the important roles of diet in health has improved. However, the enthusiasm for such products has not always been matched by the extent or quality of the evidence for their efficacy^(20,23–28)^.

Since ERG classes as a nutraceutical, it seems timely to bring together the extensive but widespread knowledge of its biology so that it may be made more widely available, and that is the purpose of this review.

## Discovery and structure

**Fig. 1.**
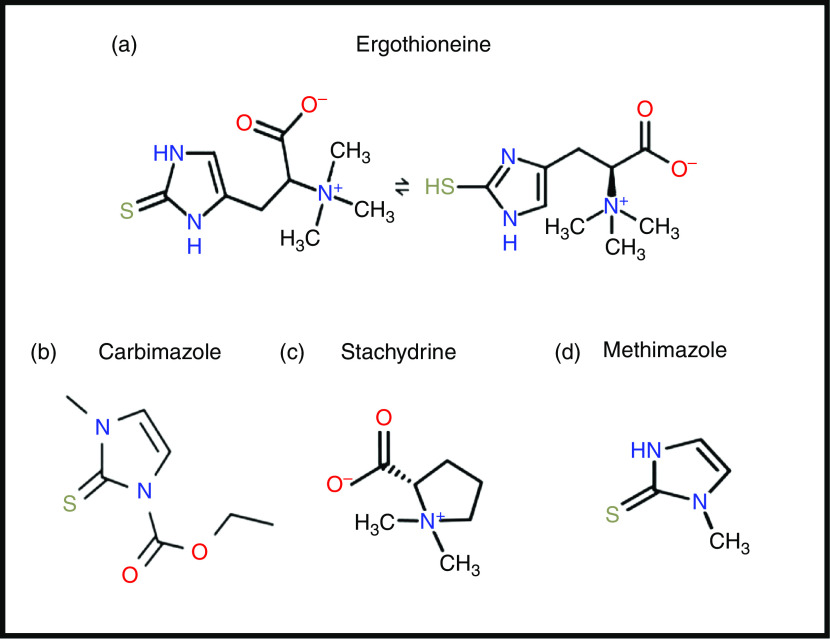
Structures of ergothioneine and related molecules. For a colour figure, see the online version of the paper.

ERG is a somewhat unusual betaine amino acid. It was discovered by Charles Tanret in 1909 while investigating the ergot fungus *Claviceps purpurea* ^(29,30)^. It is also known as 2-mercaptohistidine trimethylbetaine, and its formal International Union of Pure and Applied Chemistry (IUPAC) name is (2S)-3-(2-thioxo-2,3-dihydro-1H-imidazol-4-yl)-2-(trimethylammonio)propanoate. It is an l-histidine derivative that is N^α^,N^α^,N^α^-trimethyl-l-histidine in which the hydrogen at position 2 on the imidazole ring is replaced by a mercapto group. Its structure^(31)^, and those of some related molecules, is given in Fig. 1, indicating that is a tautomer that has both a thiol and a thione form. Although it is a thiol, and hence an antioxidant^(32,33)^, the thione tautomer is predominant at physiological pH^(34,35)^, and this makes it unusually resistant to autoxidation, i.e. simple oxidation by molecular O_2_^(32,36–38)^. Its midpoint potential for a thiol is consequently unusually high, being +0·06 V *v.* −0·2 to −0·4 V for typical thiols including glutathione^(4,39–41)^ and mycothiol^(42,43)^, and −0·193 V for the also somewhat oxidising thiol cofactor coenzyme M, which is 2-mercaptoethanesulfonate^(44)^. Its reaction with hydroxyl radicals (OH^•^) is virtually instantaneous, while it reacts only more slowly with H_2_O_2_ and/or O_2_^•−^^(38)^. Its Se equivalent is known as selenoneine and also has strong antioxidant properties^(45–52)^, but is not otherwise discussed here.

From a pharmaco-chemical point of view ERG is also unusual, since – using our standard substructure analysis^(53,54)^ in KNIME^(55)^ – we note that just two drugs marketed for human consumption (the anti-thyroxine-production drug methimazole and its pro-drug carbimazole, Fig. 1), and no endogenous genome-encoded metabolites from Recon2^(56)^ contain the imidazole-2-thione substructure^(57)^. This said, a good many fungicides do contain the benzimidazole substructure^(58)^, and a variety of benzothiazoles are used as dyes.

## Biosynthesis and phylogenetic distribution

A particular feature of ERG is that although it is more or less universally distributed among higher organisms, none of them – as is consistent with the idea that it may in fact be a vitamin requiring exogeneous sources – can in fact biosynthesise it. The chief organisms capable of its synthesis are fungi and certain yeasts^(59,60)^, though actinobacteria and certain other micro-organisms^(60–66)^, including the slime mould *Physarum polycephalum* ^(65)^, cyanobacteria^(67–71)^ and methylotrophs^(72)^ are also naturally capable of its production. The related mycothiol is typically ten times more concentrated in actinobacteria than is ERG^(73)^, and its biosynthetic pathway might provide an antitubercular drug target. Other organisms acquire ERG through transporter-mediated uptake. Thus higher plants contain it but do not biosynthesise it^(74)^; instead they and other organisms^(68,75)^ take it up from fungal production in the soil^(76–79)^, and possibly via actinobacterial^(80)^ or fungal^(80,81)^ symbionts. Animals are also considered not to biosynthesise it^(82,83)^, and accumulate it using a particular transporter, detailed below, via the plants and animals that they eat. Although not easy, it is possible to raise animals such as pigs on a diet such as casein, sucrose, lard, butter and salts that is considered to lack ERG; such animals are said to have undetectable levels of the compound^(84)^, and rats treated similarly have reproduced^(85,86)^. However, we do not know the minimum amount and its location that animals need, and these are old experiments that need to be repeated with modern techniques with lower detection limits. Only then might we have a definitive statement as to whether ERG is absolutely required as a true vitamin or not, and if so in what amounts for health. In a similar vein, ERG can be present in cell culture media and cells with organic cation transporter N1 (OCTN1)/solute carrier family 22, member 4 (SLC22A4) can accumulate it^(87)^, a fact little considered to date in cell culture studies.

**Fig. 2.**
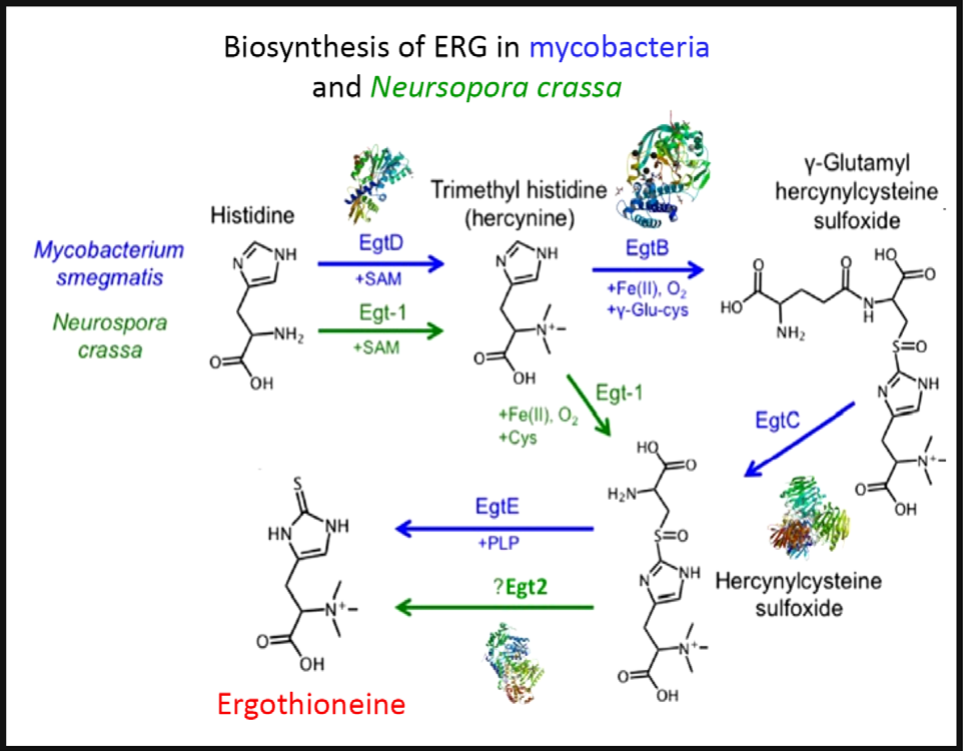
The two main pathways of aerobic ergothioneine (ERG) biosynthesis, noting the relevant enzymes and thumbnails of three-dimensional structures where known. SAM, *S*-adenosyl methionine. For a colour figure, see the online version of the paper.

To the extent that ERG is a ‘secondary’ metabolite, defined^(88)^ as a molecule whose synthesis has a relatively restricted distribution in different organisms, the biosynthetic pathways diverge from primary metabolism via the amino acids histidine, cysteine and methionine^(89–94)^. Thus (Fig. 2), histidine is trimethylated using *S*-adenosyl methionine to form trimethyl histidine, also known as hercynine^(95,96)^. This reacts oxidatively with cysteine to form hercynylcysteine sulfoxide^(97)^, which is converted to ERG. In some organisms, hercynine takes a more convoluted route via γ-glutamylhercynylcysteine sulfoxide (Fig. 2)^(94)^. Table 1 provides references for different organisms. An excellent phylogenetic analysis is given by Jones *et al.* ^(60)^. In more recent work, it has been suggested that ERG was probably first biosynthesised by anaerobes using a slightly different route that converts hercynine directly to ERG^(98–100)^, and that was later repurposed.

**Table 1.** Biosynthesis of ergothioneine in various non-recombinant micro-organisms

Organism	Selected references
*Aspergillus fumigatus*	^(257)^
*Aspergillus niger*	^(59)^
*Aureobasidium pullulans*	^(113)^
*Burkholderia pseudomallei*	^(565)^
*Chlorobium limicola*	^(99,100)^
*Claviceps purpurea*	^(105,566,567)^
*Lactobacillus casei*	^(568)^
*Methylobacterium aquaticum*	^(72)^
*Mycobacterium tuberculosis*	^(91–93)^
*Neurospora crassa*	^(89)^
*Schizosaccharomyces pombe*	^(50,569)^
*Streptomyces coelicolor*	^(255)^

**Fig. 3.**
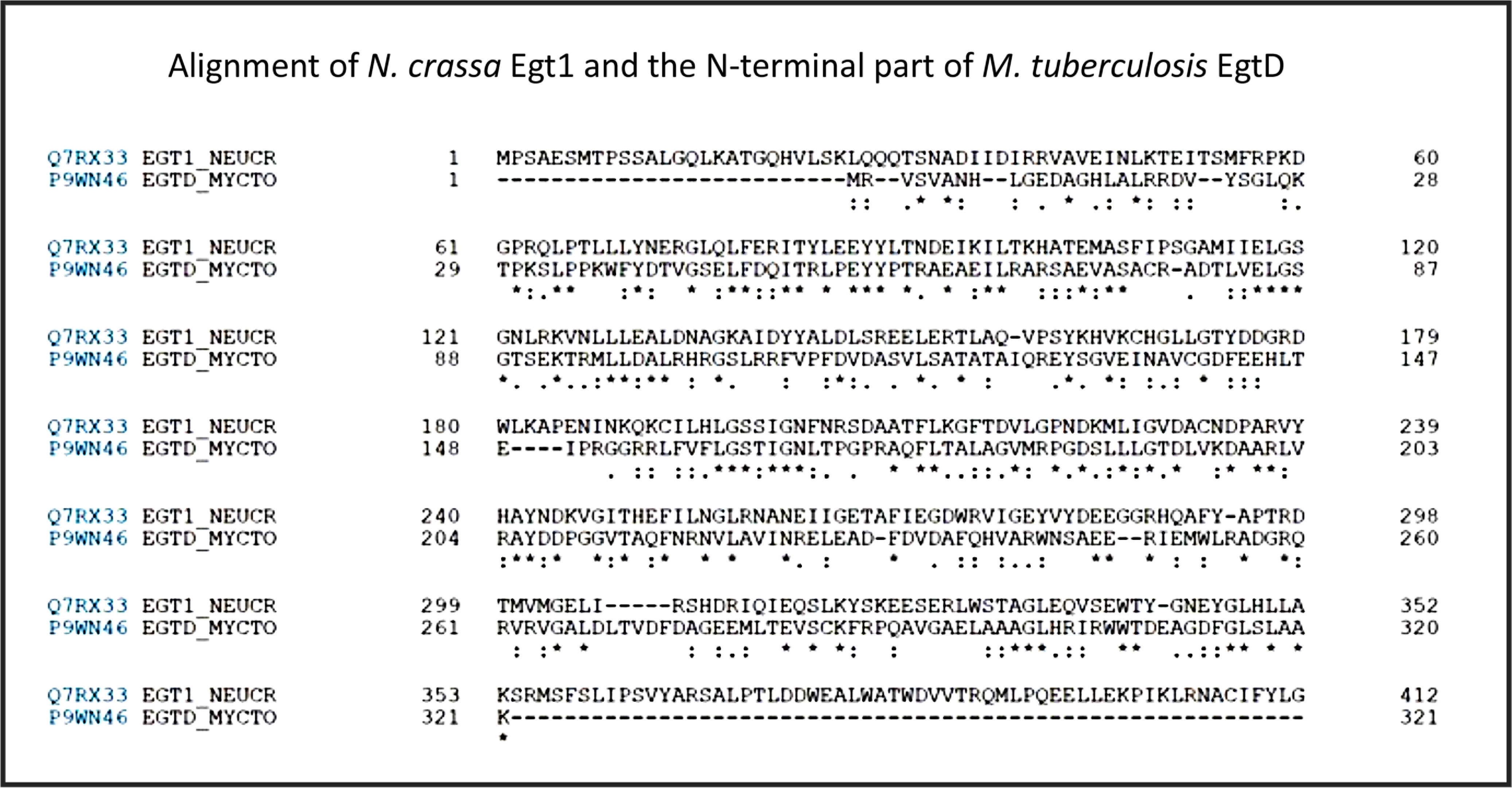
Alignment of *Neurospora crassa* Egt1 and N-terminal part of *Mycobacterium tuberculosis* EgtD. For a colour figure, see the online version of the paper.

Three-dimensional structures are known for a number of the relevant enzymes, including mycobacterial EgtB^(101)^ for example, PDB 4XBE, EgtC^(102)^ for example, PDB 4ZFJ, EgtD^(103–105)^ for example, PDB 4PIM, and *Neurospora crassa* early G1 transcript 2 (egt2) which is like egtE^(106)^ for example, PDB 5UTS. Very recently, EgtB from *Candidatus Chloracidobacterium thermophilum* was crystallised^(107)^, and engineered towards Egt1 activity. Thumbnails are given in Fig. 2. Egt1 from *N. crassa* is 876 amino acids long^(108)^, while egtD (from *Mycobacterium tuberculosis* ^(109)^) is just 321 amino acids long; since the N-terminal sequences are well conserved (Fig. 3), this implies an extra C-terminal domain catalysing the production of hercynylcysteine sulfoxide from hercynine.

In addition, enantiopure l-ERG has been synthesised chemically^(76,110–112)^, and by fermentation of genetically engineered micro-organisms (Table 2). Initial efforts in ERG synthesis were carried out in *Schizosaccahromyces pombe* using *egt1* overexpression under an inducible promoter. The N starvation and glucose starvation conditions causing long quiescence led to the maximum ERG production of 1606·3 µm while the wild-type strain produced 0·3 µm ^(50)^. *Methylobacterium aquaticum* strain 22A was engineered by expressing an additional copy of *egtBD* genes and by deleting the gene encoding histidine ammonia lyase, which degrades an ERG precursor l-histidine. The resulting strain produced up to 7·0 mg EGT/g dry cell weight and 100 μg EGT/5 ml per 7 d in test-tubes^(113)^. The filamentous fungus *Aspergillus oryzae* has also been engineered to produce ERG by expression of *egt1* and *egt2* genes from *N. crassa*, resulting in 231 mg ERG per kg of solid media^(114)^.

**Table 2.** Fermentative production of ergothioneine in recombinant micro-organisms

Organism	Genetic modification(s)	Titre	Conditions	Reference
*Aspergillus oryzae*	Expression of *egt1* and *egt2* genes from *Neurospora crassa*	231·0 mg/kg of media	Cultivation on solid, rice-based medium	^(114)^
*Escherichi coli*	Expression of *egtBCDE* genes from *Mycobacterium smegmatis*	24 (sem 4) mg/l (extracellular)	Shake flasks. Medium supplemented with yeast extract, His, Met, 20 mm-thiosulfate as sulfur source for l-cysteine synthesis. IPTG for inducing heterologous gene expression	^(115)^
*Escherichia coli*	Expression of the following genes: *egtABCDE* from *M. smegmatis*, altered *cysE* gene encoding serine acetyltransferase feed-back resistant to Cys, native *ydeD* gene encoding inner membrane Cys exporter, altered serA gene encoding l-serine Feedback inhibition-insensitive mutant of d-3-phosphoglycerate dehydrogenase. Deletion of *metJ* gene encoding transcriptional repressor	1·3 g/l (extracellular)	Fed-batch in 3-litre bioreactor, 216 h. Complex medium supplemented with IPTG, ammonium ferric citrate, pyridoxine, Met, His, and thiosulfate	^(116)^
*Methylobacterium aquaticum* strain 22A	Additional copy of egtBD expressed from a plasmid, deletion of histidine ammonia-lyase (*hutH*) gene	20 mg/l	Test-tubes. Complex medium with methanol	^(113)^
*Saccharomyces cerevisiae*		598 (sd 18) mg/l, of which 59 % was extracellular	Fed-batch fermentation in 1-litre bioreactor, 84 h. Defined medium supplemented with arginine, histidine, methionine and pyridoxine	^(570)^
*Schizosaccahromyces pombe*	*egt1* overexpression under inducible promoter	368 mg/l	N and glucose starvation. Cultivation method not given	^(50)^

IPTG, isopropyl β- d-1-thiogalactopyranoside.

Expression of *egtBCDE* genes from *Mycobacterium smegmatis* in *Escherichia coli* and optimisation of medium composition has led to 24 mg/l or 104 μm of secreted ERG^(115)^. The *egtA* gene from *M. smegmatis* was not expressed because *E. coli* contains a homologous glutamate–cysteine ligase encoded by *gshA* and involved in glutathione biosynthesis. In a follow-up study, the authors expressed *egtA* from *M. smegmatis* and it had a positive effect on ERG production. Furthermore, they enhanced cysteine and *S*-adenosine methionine biosynthesis and obtained 1·3 g/l or ERG in a fed-batch fermentation^(116)^, achieving currently the highest titre reported for heterologous ERG production.

Recently, we reported the engineering of baker’s yeast *Saccharomyces cerevisiae* for the production of ERG^(117)^. *S. cerevisiae* has a generally recognised as safe (GRAS) status and has been exploited for the commercial production of several nutraceutical compounds^(118)^; it is thus a highly attractive host for the production of ERG. We have tested sixteen different pathway variants, nine containing only fungal genes, one with bacterial genes from *M. smegmatis*, and six hybrid pathway variants containing both fungal and bacterial transgenes. The best-performing strain contained *egt1* from *N. crassa* and *egt2* from *C. purpurea*. The composition of the medium was improved using a fractional factorial design. Fed-batch cultivation resulted in 598 (sd 18) mg/l ERG after an 84-h fermentation. Some 60 % of the measured ERG was extracellular and the rest accumulated in the cells. Table 2 summarises the various recombinant expression hosts that have been used.

The distribution of solute transporters between tissues in differentiated organisms is particularly heterogeneous^(119)^, and it is to be expected that both SLC22A4 and ERG might also be distributed heterogeneously as well. This is indeed the case, their distribution being especially high in tissues that are considered to have the potential for oxidative stress^(4)^, such as erythrocytes^(120–129)^, bone marrow^(130)^, liver and kidney^(85,131)^, seminal fluid^(132,133)^ and the lens and cornea of the eyes^(134)^. It may also be accumulated in the CNS^(135,136)^.

Finally, here, we note – as with the activity of the ‘master Fe regulator’ hepcidin^(137–141)^, that acts chiefly via the ferrous Fe transporter ferroportin – that the action of a transporter in concentrating a substance in one tissue will typically lead to its depletion from another. Consequently, it is necessary to measure all relevant compartments to assess whether a molecule such as ERG, whose distribution is strictly transporter-mediated, is protective against a particular disease/effect or otherwise in a particular place or case.

## SLC22A4: the ergothioneine transporter

Although this view remains controversial, even hydrophobic molecules do not normally ‘float across’ whatever phospholipid bilayer portion of cells may be untrammelled by proteins. Xenobiotics in particular need to ‘hitchhike’ on protein transporters that have presumably evolved for ‘natural’ substrates but that are capable of their uptake^(142–152)^. While transporters seem to have remained somewhat understudied^(153)^, those transporters involved in uptake and encoded by the human genome are now catalogued formally as SLC for solute carriers^(154,155)^, with efflux transporters mainly being classed as ABC families^(156)^.

One solute carrier, previously known as organic cation transporter N1 (OCTN1)^(157,158)^, and now known as SLC22A4 (the human version is Uniprot Q9H015), a 551-amino-acid transporter with three glycosylation sites, is of special interest. It had been designated as a transporter of carnitine and of the (non-physiological) tetraethylammonium cation. However, in a really groundbreaking paper, Gründemann *et al.* ^(130)^ recognised that the rates observed (using radioisotopes) were too small to be physiologically meaningful, and using a method that we would now refer to as ‘untargeted metabolomics’^(159–164)^, they incubated two kinds of HEK293 cells in serum. The first were normal cells, that, as with many transporters^(119)^, do not in fact express SLC22A4 at significant levels, while the second had been engineered to overexpress the transporter. They then simply looked for those molecules that were most differentially taken up, a molecule called stachydrine, also known as proline betaine, being the main one observed, Stachydrine is a constituent of citrus juices^(165–167)^. Some elementary cheminformatics based on structure similarity searches^(57,168)^ indicated that ERG, as a betaine, was indeed similar to stachydrine. Incubating the cells just with ERG showed that it was taken up about 100 times more quickly than was tetraethylammonium, leading to the designation of SLC22A4 as ‘the’ ERG transporter^(130)^. Subsequent work^(87,169–172)^ has confirmed and reinforced this view of SLC22A4 and its homologues^(173)^ as having significant specificity for ERG, and weak activity for various drugs^(174–177)^. It is concentrative, coupled in humans to influx of 2 or 3 Na^+^ ions per ERG transported^(130)^. Interestingly, it is up-regulated chronobiologically just before meal times^(175)^. The rat and human orthologues are interchangeable^(178)^. Tissue levels of ERG depend on an exogenous supply^(179)^, but are then well correlated with the expression levels of SLC22A4^(3,180)^. SLC22A4 expresses well even in microbial systems^(181)^, and is widely tolerant of amino acid substitutions^(182)^. As yet, no other transporter with significant activity for ERG in humans is known, making it a potentially interesting drug target^(183,184)^.

### Expression patterns

SLC22A4 is known to express in the intestinal lumen^(185)^, acting to take up ERG, as well as some xenobiotics including pyrilamine, quinidine and verapamil, and having multiple known but weak inhibitors.

**Fig. 4.**
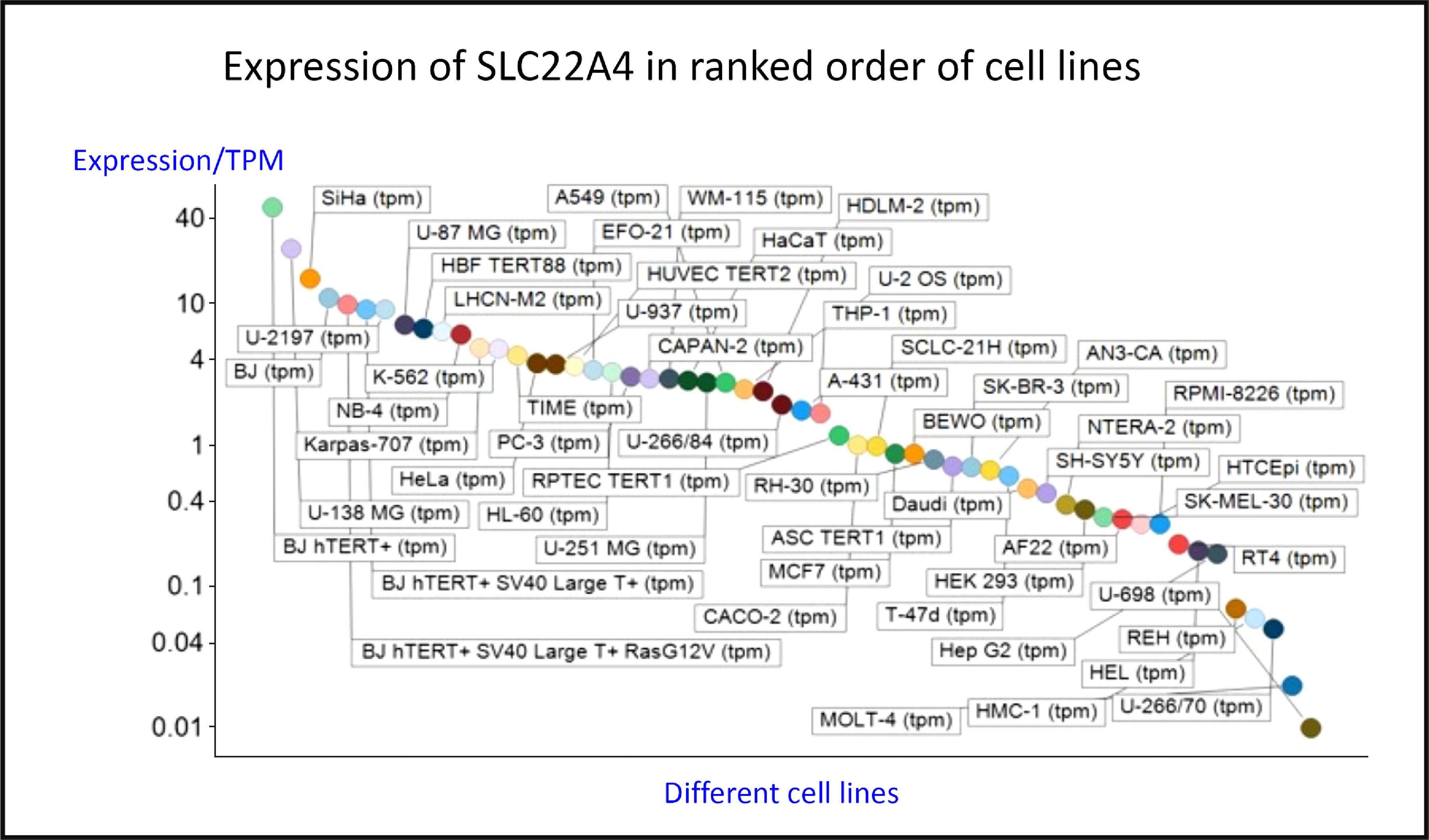
Differences in expression of SLC22A4 transcript in a series of mammalian cell lines. Data are from Thul *et al.*^(186)^ and O’Hagan *et al.*^(119)^. For a colour figure, see the online version of the paper.

Fig. 4 shows the expression of the transcript for SLC22A4 in fifty-six cell lines using previous data^(119)^ taken from the human protein atlas^(186)^, indicating a range in expression levels between different cell lines of more than 4000-fold, a number not atypical for human transporters^(119)^. Tissue expression data are given in Fig. S4 of O’Hagan *et al.* ^(119)^.

The intracellular expression patterns are as yet uncertain, with early claims for a mitochondrial expression^(86,187–190)^ being based on very weak and contradictory evidence^(8)^. However, while the cellular uptake of ERG does require plasma membrane expression, the latest version of the protein atlas indicates mitochondrial expression as well^(191)^. However, as is well known, antibody specificities are rarely either known or absolute^(192–198)^. Thus, relying on antibody evidence alone is rather hazardous, and, as mentioned before^(8)^, mitochondrial transporters have an SLC25 family designation^(199,200)^. Definitive measurements on the uptake or otherwise of ERG into isolated mitochondria, or indeed into other organisms that cannot make it, are eagerly awaited.

### Evolution and phylogenetic distribution of SLC22A4

Phylogenetic analyses^(201,202)^ indicate that homologues of SLC22A4, a relative of the major facilitator superfamily 2, exist only in vertebrate animals, especially mammals, birds and fish, with occasional examples in reptiles (for example, *Xenopus* spp.).

In practice, it appears that the transporters responsible for the uptake of some 85 % of pharmaceutical drugs actually evolved to take up exogenous natural products^(203)^. In the case of the cocaine transporter^(204)^, a simple narrative can serve to explain how a cocaine-mediated ability to outrun a predator such as a sabre-tooth tiger can rather obviously be selected provided the bioactive substance is actually taken up by the host. More generally, the ability to transport exogenous natural products is likely to be selected for when these confer fitness benefits on the host^(205)^, and this probably underpins the finding that successful, marketed drugs are indeed similar to (mainly ‘secondary’) natural products^(203)^.

## Oxidative stress

**Fig. 5.**
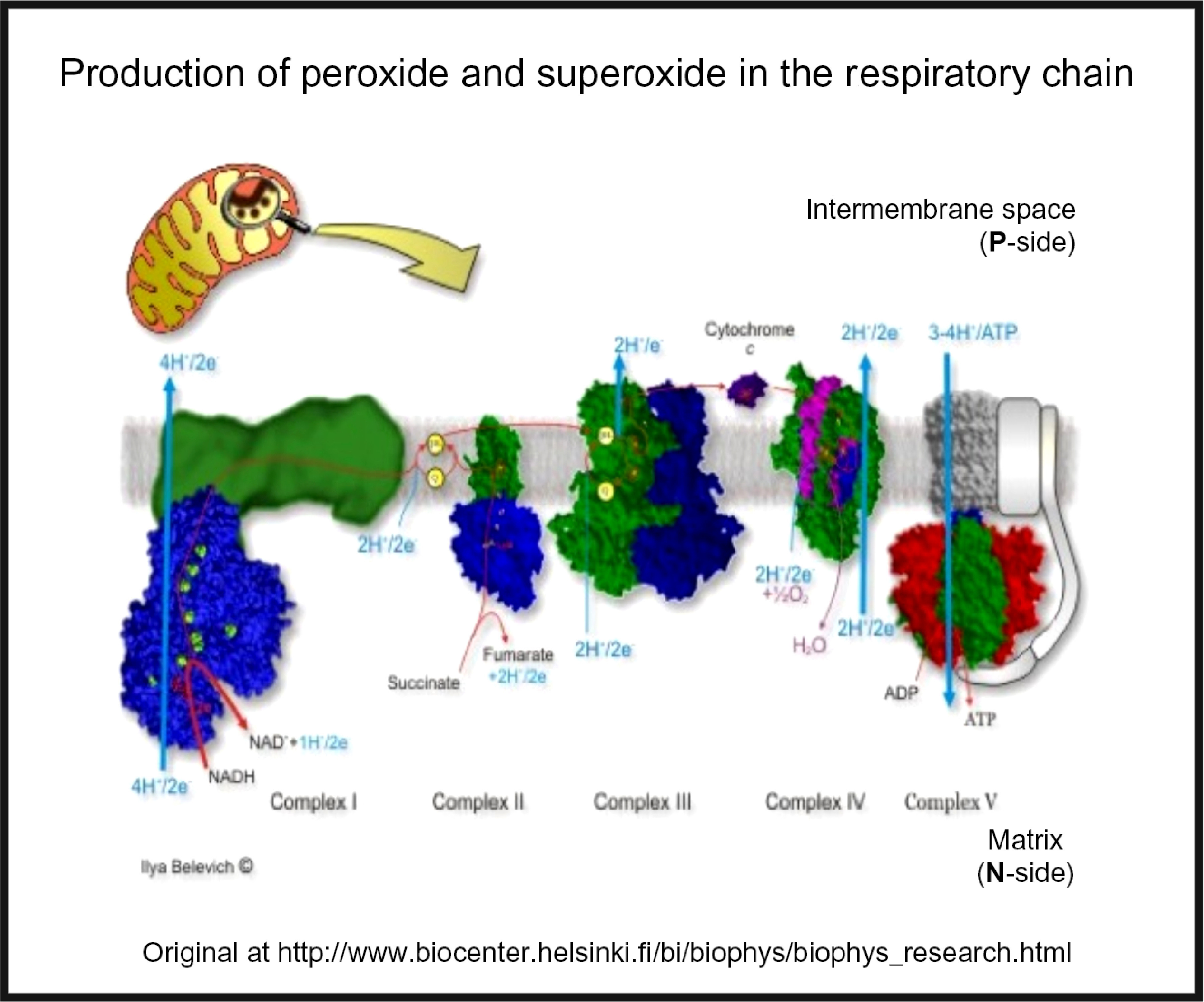
Superoxide and peroxide are produced by 1- and 2-electron reduction of dioxygen by the mammalian respiratory chain. For a colour figure, see the online version of the paper.

Oxidative stress is widespread to the point of ubiquity in chronic, inflammatory diseases^(206,207)^, with over fifty papers having the words ‘oxidative’, ‘stress’ and ‘review’ in their titles at PubMed in 2018 alone! It can occur when oxygen tension is low and respiratory chains are over-reduced such that they reduce O_2_ with one electron to superoxide or two electrons to H_2_O_2_, instead of the four that are used during the reduction of dioxygen to water by cytochrome oxidase^(208)^ (Fig. 5). Peroxides are also produced *in vivo* by various oxidases and peroxidases, such as xanthine oxidase, by reduction of dioxygen (for example, Babior^(209)^, Cave *et al.* ^(210)^ and Bedard & Krause^(211)^).

**Fig. 6.**
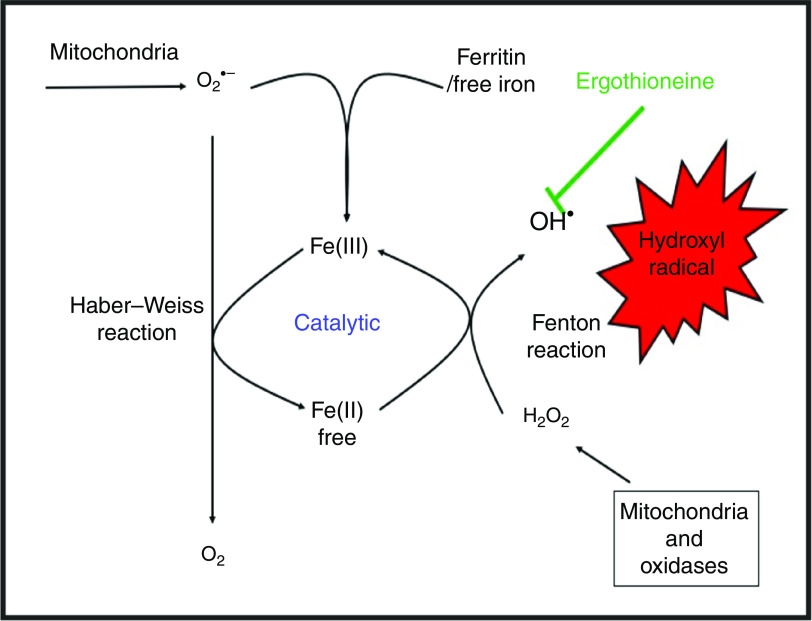
Catalytic roles of unliganded iron in hydroxyl radical production via the Fenton and Haber–Weiss reactions. This can be stopped by ensuring that iron is fully liganded. For a colour figure, see the online version of the paper.

While H_2_O_2_ and superoxide are certainly capable of effecting unwanted oxidations, it is the hydroxyl radical that is the key. Thus an important reaction of H_2_O_2_ with (free or poorly liganded) Fe(II) is the Fenton reaction^(208,212,213)^, leading to the very reactive and damaging hydroxyl radical (OH^•^):(1)
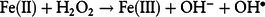



which can react within nanoseconds with anything adjacent. The role of Fe is absolutely vital here^(208,213)^. Superoxide can also react with ferric Fe in the Haber–Weiss reaction^(214–216)^ to produce Fe(II) again, thereby effecting redox cycling, and meaning the ‘iron’ is catalytic (Fig. 6):(2)
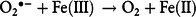



In addition O_2_^•−^ can release ‘catalytic’ Fe from Fe-S clusters in certain proteins and from ferritin^(208,217)^, another way in which it can promote the Fenton reaction. Note that other reactions can produce OH^•^ anaerobically^(218)^. Because OH^•^ is so reactive it is not really observable in its free form; its action is detected via products of molecules with which it has reacted. These include 8-oxo-guanine derivatives^(219)^, nitrotyrosine^(220–222)^ (itself formed from peroxynitrite^(223,224)^, possibly formed more commonly via superoxide^(225,226)^), 4-hydroxy-nonenal^(227)^, and many others reviewed previously^(208)^. In evaluating the antioxidant potency of ERG or anything else, it is molecules such as these that are normally assessed. Although the literature is somewhat scattered and heterogeneous, it seems clear that as well as hydroxyl radicals^(228–232)^, ERG can also react with and detoxify, or prevent the formation of, singlet oxygen^(233–242)^, ozone^(243)^, superoxide^(231,241,244–246)^, peroxide^(32,124,247,248)^, hypochlorite^(32,232,249)^ and peroxynitrite^(224,231,250,251)^. Consequently, it is a potent antioxidant.

## Roles in the producer

Although it is not *a priori* certain that they would be the same in both producer and consumer organisms, it is of interest, before looking at higher organisms, to consider the roles of ERG in the producer organisms themselves. In the case of *C. purpurea*, the ERG serves as an antioxidant to neutralise a plant host defence response based on H_2_O_2_ that would otherwise inhibit the production of its conidia^(252,253)^. In *M. tuberculosis* and other mycobacteria^(254)^, and also in other actinobacteria^(255)^ and in fungi^(247,256,257)^, it is clear that ERG can have a role as an antioxidant^(66,258–260)^ and also act as a buffer against reductive stress^(261)^. In nature many organisms can be subjected to oxidative stress, and produce a variety of molecules to combat it^(262–270)^. This also seems true of mushrooms^(271,272)^, where ERG is typically the main antioxidant^(273–275)^, and where it may also inhibit the oxidative enzyme tyrosinase^(276)^. Given suggestions that the ‘purpose’ of secondary metabolite formation is to serve as a signalling molecule in different cells of the producer organism, i.e. as a pheromone^(277)^, it is interesting to note that this may also involve crosstalk of ERG^(37)^, due in part to the complex networks in which it may be embedded^(278)^. The same is true of the imidazole thiol-containing ovothiol^(279,280)^. In a similar vein, and although outwith our scope here, we note the potential of other antioxidant natural products such as curcumin^(281–286)^.

## Nutritional sources

Betaines are generally seen as nutritionally beneficial^(287)^, and many are ‘compatible solutes’^(288–293)^, defined as solutes whose accumulation assists the survival of the organism when undergoing various kinds of stress such as osmotic or thermal stress. However, of these, only ERG is seen as a major antioxidant. Although a variety of foodstuffs such as oats^(294,295)^ contain ERG because they take it up from exogenous sources, it is really mushrooms that are the prime sources for humans^(18,294)^. Indeed, ERG has been proposed as a nutritional biomarker for mushroom consumption^(296,297)^, albeit that different mushrooms typically contain different amounts^(275,298–300)^, and these can vary with physiological or environmental conditions^(301–305)^. Those with the highest amounts include oyster mushrooms (*Pleurotus* spp., up to 4 mg/g DM)^(306)^, the golden oyster *Pleurotus citrinopileatus* with 10·65 mg/g DM^(307,308)^, and shiitake (*Lentinula edodes*, about 1 mg/g DM), while of those more common outside Asia, porcini or ceps (*Boletus edulis*, > 7 mg/g DM), stand out^(6,294,300)^. However, even common field or ‘button’ mushrooms, *Agaricus bisporus*, contain some 0·4 mg/g DM^(275,299,300,309)^. Note too that tempe(h), the result of a solid substrate *Rhizopus oligosporus* fermentation^(310–314)^, also contains high levels of ERG^(6)^. Mushrooms may also be a significant benefit to those seeking a meat-free diet as they can be made to share certain organoleptic features with meat^(315,316)^. Notably, ‘the production of cultivated, edible mushrooms worldwide has increased more than 30-fold since 1978, whereas the population has only increased by about 1·7-fold during the same period’^(10,317)^.

Some studies that have demonstrated nutritional/health benefits of mushrooms and their antioxidant activity^(125,271,318–351)^) did not always seek to deconstruct these into their constituents such as ERG, but ERG is clearly the chief mushroom antioxidant. We note too that some effects may be dependent on the composition of the gut microflora^(352)^, that are of course themselves likely to be changed by ERG, just as they are by many other non-antibiotic drugs^(353)^.

## Safety

Producer organisms such as mushrooms are well known to make many secondary metabolites, some of which can be highly toxic^(354–356)^ and by various mechanisms^(357)^. Notwithstanding the highly variable intake between individuals^(358)^, however, a number of high-dose studies have indicated that ERG is safe for mammalian consumption at levels far in excess of those likely to be encountered in foodstuffs^(125,131,359,360)^, and it has been declared safe by relevant committees such as those of the European Food Standards Agency^(361,362)^. It also lacks any detectable mutagenicity or genotoxicity in such assays, even at very high doses^(363,364)^.

## Analytics

Leaving aside early efforts such as the colorimetric methods of Hunter^(365)^, of Melville and colleagues^(76,85,366)^ and of Carlsson *et al.* ^(367)^, a variety of analytical methods have been proposed^(4)^, mostly involving capillary electrophoresis^(368,369)^ or chromatography^(368,370–372)^ coupled to absorbance^(373,374)^, fluorescence detection^(375–378)^, electrochemical detection^(379)^ or MS^(72,127,256,368,378,380–382)^. A useful feature is that ERG is unusually stable, in that anhydrous ERG decomposes only at 275–276°C^(76)^, allowing its isolation at temperatures close to that of boiling water^(72)^. As judged by the reversibility of its acid–base titration^(383)^, it is also stable to extremes of pH.

Industrial purification of glycine betaine is done by extraction with water^(384)^ and subsequent ion exchange chromatography^(384,385)^, which can be done in simulated moving bed fashion^(386)^. Glycine betaine can then be crystallised^(384)^. As glycine betaine is structurally similar to ERG, this straightforward industrial process could potentially be adapted for ERG.

## Serum and other concentrations

While most ERG is inside erythrocytes in whole blood^(6,121,122,129,387)^, there have been a number of measurements of ERG concentrations in serum. Unsurprisingly it varies with diet^(388,389)^, starvation^(390)^, age^(391,392)^ and other factors, including diseases of oxidative stress^(393)^, with typical values of 20–100 µg/ml. A detailed list is provided by Cheah & Halliwell^(4)^; a smaller listing is given in Table 3. Interestingly, ERG is also present in seminal fluid^(394–396)^ and human breast milk^(6)^. Any possible correlation with male fertility^(397)^ seems not to have been established, though there were no negative effects^(398)^, and ERG improved oocyte quality and maturation in cows and sheep^(399)^. ERG is also present in eye lens, where its concentration is lower in individuals with cataracts^(400)^.

**Table 3.** Concentrations of ergothioneine in human serum

	Concentration		Study
Crohn’s disease	7 µg/ml		^(401)^
Healthy volunteer	38 µg/ml		^(401)^
Healthy 1–10 years	15–20 µg/ml		^(387)^
Healthy 11–18 years	37 µg/ml		^(387)^
Healthy 19–50 years	23–30 µg/ml		^(387)^
Healthy middle-aged and older	Median 1 μm = 229 ng/ml, range 0·36–3·08 μm*	Inverse correlation with age	^(571)^
Mice on normal diet	58 µg/ml		^(131)^

* Molecular weight = 229·3, so 1 mm = 229 mg/l.

## Metabolism and excretion

ERG is metabolised and excreted only slowly^(360,371,401,402)^. In a recent and detailed study, Cheah *et al.* ^(360)^ administered 5–25 mg daily doses of ERG to human volunteers for 7 d. There was little urinary excretion (<4 %), and the main metabolites were hercynine, plus lesser amounts of *S*-methyl-ERG, whose concentrations were well correlated with the level of ERG and the dose of ERG given. Similar observations were made in mice^(131)^. Various other biomarkers of oxidative stress (for example, 8-iso-PGF2α from lipid peroxidation) were lowered concomitantly in the human study, attesting to the antioxidant functions of ERG *in vivo*, although in this case the healthy young subjects were probably not suffering from oxidative stress. There was also quite some variation in uptake between individuals, presumably reflecting variation in their expression of SLC22A4. *Agrobacterium radiobacter* ^(403)^ and other bacteria^(404–409)^ contain an ergothionase that degrades ERG to thiolurocanic acid (3-(1H-imidazol-5-yl)prop-2-enethioic S-acid) and trimethylamine, also implying that such cells possess one or more transporters for ERG. The thiolurocanic acid can be further degraded to glutamate^(410)^.

## Apparent fitness benefits and bioactivities of ERG and the role of SLC22A4

Given the great technical difficulties associated, because of its ubiquity, with withholding ergothoneine from a human or animal diet, one means of ‘removing’ ERG from a host is to remove the ERG transporter by genetic means. This has in fact been done in mice^(401)^; such SCL22A4^–/–^ mice had immeasurably low levels of ERG relative to controls, and were much more sensitive to oxidative stress than were the wild type. Similar effects were observed in *Caenorhabditis elegans* ^(411)^. Polymorphisms in SLC22A4, of which there can be many^(177,412–415)^, under selection^(416)^, have also been associated with ischaemic stroke^(417)^, erythroid differentiation^(418)^, hearing loss^(412)^, rheumatoid arthritis^(126,180,419–427)^, lupus^(428)^, Crohn’s disease^(401,429–436)^, hearing loss^(412)^, type 1 diabetes^(437)^ and diabetic embryopathy^(438)^. The expression of SLC22A4 can itself be modulated by other factors, including by PPAR-α activity^(439)^. The very diversity of these diseases speaks naturally to a broad and common underlying cause, the easiest of which involves mechanisms of oxidative stress, inflammation and cell death.

## Mechanisms of action

It has become common to discover a binding of a small molecule to another molecule such as a protein, and assume that this interaction, leading to activation or inhibition of the target, constitutes ‘the’ mechanism of action of the small molecule at a physiological level. Unfortunately this is rarely the case, and known drugs, despite often being selected for inhibiting potently a specific molecular target^(147)^, have, on average, six known binding targets^(440)^. When these interactions ramify through a complex and non-linear biochemical network it can be hard to apportion the effects of a small exogenous molecule between the various interactions^(441–443)^.

**Fig. 7.**
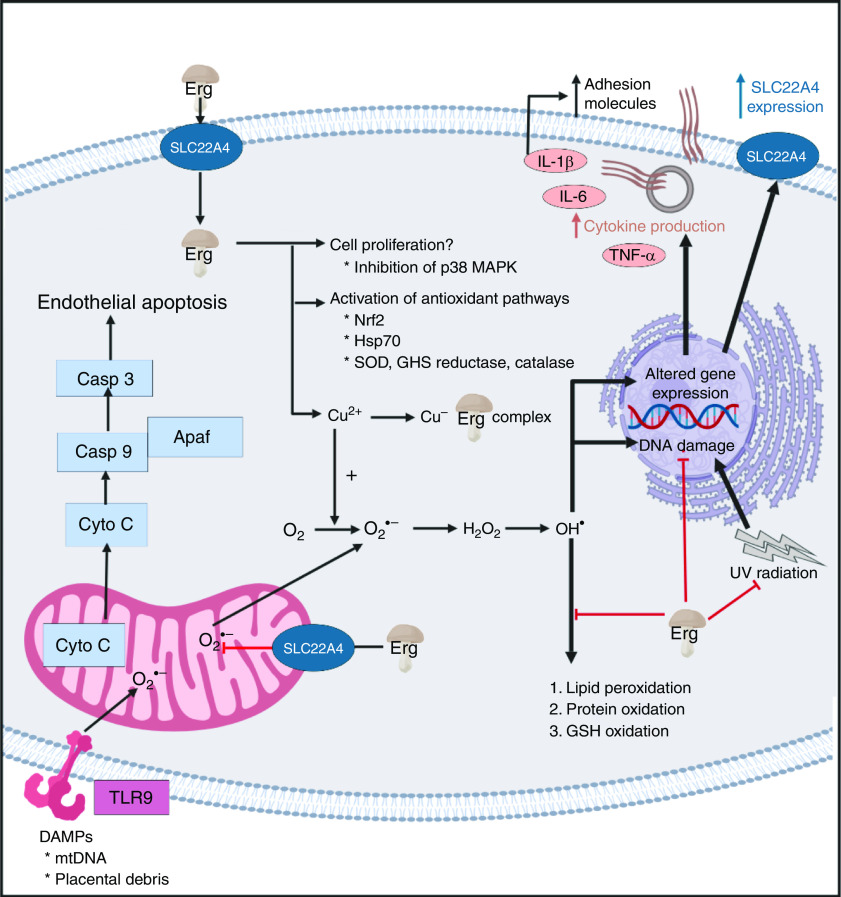
Overview of some of the effects of ergothioneine in mammalian systems. For a colour figure, see the online version of the paper.

A standard view of systems or network biology (for example, Kell^(444)^ and Kell & Knowles^(445)^) develops these ideas in four stages. In stages 1 and 2 we simply recognise the actors and the interactions between them at a qualitative level. Stages 3 and 4 then seek to describe the equations reflecting individual steps and the values of the parameters of those equations. Armed with these we can make an ordinary or, if spatial resolution within a compartment is required, a partial differential equation model of the system. This can then be run and the sensitivities of each step determined^(446–448)^. We are very far from this last part, and so studies of the effects of ERG have in general^(449)^ been rather descriptive in nature. Many have been at the level of processes rather than mechanisms, and they have been reviewed in detail^(3,360)^. Table 4 and Fig. 7 provide a selection of determinands that have been shown to change their concentrations or activities when ERG is added to the system of interest. In many cases it is not at all clear what the proximate mechanisms are. Note as just one example that the highly promiscuous transcription factor NF-κB^(450–452)^, whose frequency-dependent activity directly affects the expression of hundreds of enzymes^(453,454)^, is itself redox-sensitive^(455–458)^, and is affected by ERG^(459,460)^, while NF-κB increases the rates of SC22A4 transcription^(419)^. Thus, deconstructing the many possible direct and consequential interactions of ERG with proteins, *v.* whether these are simply a consequence of its provision of a more reducing environment, is likely to be a formidable task. In a similar vein, the effects of ERG on the microbiomes of the hosts are likely to be significant, but do not yet seem to have been studied.

**Table 4.** Biological properties whose expression or activity varies on exposure of a biological system to ergothioneine (ERG) or a modulation of SLC22A4 activity

Determinand	System	Comments	Selected reference(s)
Cataract formation induced by glucocorticoid	Developing chick embryo	ERG inhibits	^(572)^
Cell death	Human neuronal hybridoma cell line N-18-RE-105	H_2_O_2_ challenge	^(251)^
	*Caenorhabditis elegans*	Protection from amyloid-β-induced cell death	^(521)^
Cell injury	Rat pheochromocytoma cells	Methylglyoxal challenge	^(573)^
Cell proliferation	K562 cells	Involvement of SLC22A4	^(418)^
	Caco-2 cells	Involvement of SLC22A4	^(429)^
Diabetic embryopathy	Rats	ERG reduced it to control levels	^(574)^
DNA damage in mitochondria	HeLa, RAW 264·7, HaCaT, PC12 cells	siRNA knockdown of SLC22A4	^(3)^
Embryo development	Sheep	Improvement, despite non-uptake of ERG	^(399,575)^
Embryo quality and maturation	Cows	Improvement	^(576)^
Excitotoxicity caused by *N*-methyl-d-aspartate	Rat	Protection by ERG	^(577)^
Glycolysis	Erythrocytes	Preservation of lactate production during starvation	^(578)^
Hepatocyte injury induced by CCl_4_	Hepatocytes	Protection, also by β-hydroxy derivative	^(579)^
Immune modulation	Mouse macrophages	Skewing towards a Th17 response	^(580)^
Immunotherapy	Tumour cells	Improved vaccine responses by dampening cytotoxic T-lymphocyte suppression	^(581)^
IL-8	Alveolar macrophages	H_2_O_2_ and TNF-α induction. Possible intermediacy of NF-κB	^(460)^
Fe incorporation into protoporphyrin	Erythrocyte fractions	Said to keep Fe reduced; does not seem to have been confirmed	^(562)^
Kidney fibrosis	Mice	Worsens during chronic kidney disease if SLC22A4 removed	^(582)^
Lipid peroxidation	HeLa, RAW 264·7, HaCaT, PC12 cells	siRNA knockdown of SLC22A4	^(3)^
	*In vitro*	Free radical initiated with anthracyclines	^(583)^
Lung injury	Rats	Cytokine treatment; damage prevented by ERG	^(505)^
Memory	C57BL/6J mice	Attenuates memory loss induced by d-galactose; synergistic with melatonin	^(584)^
Metal ion chelation	Co^++^, Cu^++^, Ni^++^, Zn^++^	Direct and within enzymes	^(527)^
	Cu^++^>Hg^++^>Zn^++^> Cd^++^>Co^++^>Zn^++^	IR measurements	^(585)^
	Cu^++^	NMR	^(586)^
	Cu^++^	Chelation prevents DNA damage	^(473)^
	Cu^++^	Chelation prevents DNA damage	^(472)^
	Hg^++^	In intact erythrocytes, after glutathione	^(587)^
Mutagenesis protection	Multiple	Often involving singlet oxygen	^(588–591)^
Neuronal differentiation	Neural progenitor cells	ERG stimulated differentiation	^(592)^
NF-κB	MH7A cells	Affects SLC22A4 expression	^(419)^
Nrf2	HaCaT skin cells	Anti-apoptotic following UV irradiation	^(549)^
*S*-nitrosoglutathione catabolism	*In vitro*	ERG stimulates	^(593)^
S6K1 mTOR and neurotrophin 4/5-TrkB	Neural stem cells	Rapid induction after ERG exposure	^(594)^
Sickle cell anaemia		ERG is protective	^(595)^
SIRT1 and SIRT6	Endothelial cells	Protection *v.* glucose-induced senescence	^(482)^
Sperm motility	Boars	Protection *v.* Cu^++^ inhibition	^(596)^

siRNA, small interfering RNA; mTOR, mammalian target of rapamycin; SIRT, sirtuin.

It seems clear that the chief role of ERG, via a variety of mechanisms, including directly, is to serve as an antioxidant and cellular protectant against various kinds of reactive oxygen and N species.

### Cytoprotection

At a high level, ERG is seen as an excellent cytoprotectant against all kinds of cellular insults^(3,4,6,124)^. We split some of the more detailed analyses into subdivisions in the following few sections.

### Oxidative stress

Oxidative stress can be defined and measured in many ways^(461–468)^, but is broadly taken to involve a dysregulation in the various redox systems of the organism of interest, coupled to the production of various ‘reactive oxygen species’, principally peroxide, superoxide, hydroxyl radical, and singlet oxygen. ERG has been shown to decrease oxidative stress in the liver and kidney of rats^(469)^, rescued cells from β-amyloid-induced apoptotic death^(231)^, protected against palmitic acid-induced cell death^(470)^, mercuric chloride-induced cellular dysfunction^(471)^, and prevented Cu-induced oxidative damage to DNA^(472,473)^. It is protective against the oxidation of various kinds of molecule^(251,474)^, including astaxanthin^(475)^, and accumulates in a guinea-pig model of non-alcoholic fatty liver disease^(476)^, massively so in mouse models of myocardial infarction and heart failure^(477)^, and in a rat model of optic nerve crush^(478)^. It serves to resist H_2_O_2_-induced cell death^(479)^, pyrogallol-induced toxicity^(124)^, cisplatin-^(480)^ or oxaliplatin-induced^(481)^ toxicity, glucose-induced senescence^(246,482)^, as well as lipopolysaccharide-induced inflammation^(483)^. In particular, it is protective against ischaemia–reperfusion injury^(484–486)^, and may have uses in prolonging the lifetime of stored blood^(487)^. Probably such antioxidant activities are at the core of its biological benefits.

## Ergothioneine as a therapeutic for chronic inflammatory diseases?

Inflammation and oxidative stress go hand in hand^(3)^, since reactive oxygen species (and materials such as bacterial cell wall components that can lead to their generation^(206,488)^), lead to the production of inflammatory cytokines. Although a great many chronic, inflammatory diseases are recognised as having an oxidative stress component^(223)^, the history of treating them with antioxidants such as ascorbate has largely been a litany of failure, with the treatment arm often even giving worse prognoses than the placebo^(6,223,489–501)^. Arguably this is because nominally antioxidant molecules such as ascorbate have complex, multi-electron redox chemistry, and can in fact act as pro-oxidants^(502,503)^, especially in the presence of free Fe^(208,213)^ or Cu^(504)^. This is not an issue with ERG, however, partly because it can chelate them, and ERG levels are decreased, or ERG has been proposed as a useful antioxidant, in diseases such as acute respiratory distress syndrome^(505)^, CVD^(506,507)^, chronic obstructive pulmonary disease^(223)^, pre-eclampsia^(8)^ (see also Turner *et al.* ^(128)^), overhydrated hereditary stomatocytosis^(508)^, and is significantly lowered in others such as certain leukaemias^(121,122)^. The evidence for this comes from a variety of sources, including metabolite measurements in human subjects^(121,122,509)^, and intervention studies in both animals^(505)^ and cell lines^(3,124,506)^. In particular, there is a notable relationship between ERG consumption and longevity (Fig. 6 in Beelman *et al.* ^(10)^), while in a 3236-participant Swedish study, ERG was the metabolite most strongly connected to a ‘health conscious food pattern’ and was associated with a lower risk of coronary disease (hazard ratio (HR) per 1 sd increment of ERG, HR = 0·85; *P* = 0·01), cardiovascular mortality (HR = 0·79; *P* = 0·002) and overall mortality (HR = 0·86; *P* = 4 × 10^–5^)^(509)^.

### Neurological diseases and cognitive function

Mushrooms have been shown to have very substantial effects on cognitive function^(341,348,510–513)^, and this is mainly ascribed to their ERG content, that also deceases with the age of the consumer^(391)^. The kinds of evidence include both double-blind, placebo-controlled clinical trials^(341)^ and observational (cross-sectional) studies in both humans^(348,510–512)^ and rodents^(513)^. Thus, consuming 1·5 mushroom servings per week was associated with a halving of the incidence of mild cognitive impairment (a precursor of Alzheimer’s dementia), while intake of nine servings per week was associated with a five-fold decrease^(348)^. Note, however, that at least one mushroom trial indicated no measurable benefits in healthy young physical education students^(514)^. Brain and serum ERG levels are also markedly different in Parkinson’s disease^(515)^, reviewed in Hang *et al.* ^(516)^, Shao & Le^(517)^ and Shah & Duda^(518)^, and even in sudden infant death syndrome^(519)^, and ERG has been shown to be protective against β-amyloid-induced neuronal injury^(520)^ and cytotoxicity^(521)^. It can also act as an antidepressant^(522)^. The evidence for this comes from direct studies^(520)^ and feeding experiments^(522)^ in mice, as well as via the reduction of β-amyloid peptide in a transgenic *C. elegans* model^(521)^. As mentioned above, SLC22A4 polymorphisms are associated with ischaemic stroke^(417)^.

## Use of ergothioneine as an antioxidant in processed foodstuffs

Just as living beings exploit antioxidants, most foodstuffs can also be oxidised to produce taints, rancidity or other undesirable products^(523–525)^, often via the Fenton reaction^(208,526)^. ERG inhibits polyphenoloxidases^(527)^, and thus ERG has been used in the feed of the shrimp *Marsupenaeus japonicas* to prevent melanosis during storage^(528)^, while ERG-rich mushroom extract has also been used to prevent melanosis in post-harvest storage of the crab *Chionoecetes japonicus* ^(529)^. Thus, one can also envisage a role for ERG, whether natural or added, in extending shelf lives and commercial value^(245,328,475,528–539)^. The thermostability of ERG is of particular importance here.

## Use of ergothioneine in cosmetics

Just as processed foodstuffs ‘age’, so do tissues such as the skin, and although the same principles apply^(540)^, it is common to refer to nutraceuticals that are also aimed at having cosmetic benefits as ‘cosmeceuticals’^(541–543)^. Here too, ERG has been widely used^(543–547)^, since much skin damage is caused by UV-mediated reactive oxygen species production^(548)^; indeed, ERG is known as a skin protectant^(240–244,549–551)^.

## Role of ergothioneine as a cofactor?

Although it is possible that the role of ERG lies simply in being an antioxidant capable of mopping up hydroxyl radicals and other reactive oxygen species, especially in prokaryotes^(36,66,93,254,255,258–260,552,553)^, the roles of most other vitamins involve interaction with proteins, often as cofactors. This is also true for mycothiol^(73,554–556)^, though that molecule can also serve as a signal and nutrient resource^(557)^. However, despite many hypotheses^(558,559)^, the only example of ERG acting as a cofactor known to date is an involvement in the biosynthesis of lincomycin^(560,561)^. An early paper^(562)^ implying an involvement of ERG in the maintenance of a reduced state of Fe in Hb, although apparently accurate, does not seem to have been followed up to date.

## Conclusions

There is increasing awareness that health may be enhanced via the consumption of substances that either have no recommended daily intake or are taken at levels greater than normal, and such substances are commonly referred to as nutraceuticals. ERG, a potent and effective antioxidant, seems to be an important nutraceutical, and we rehearse a very broad literature, involving a great many cells, tissues and organisms, to that effect. The chief source of ERG in the human diet is mushrooms (usually the fruiting bodies of Basidiomycetes). The fact that a specific transporter known as SLC22A4 has evolved and been selected to effect ERG uptake in all known animals suggests strongly that ERG is of benefit to its consumers. While the evidence that ERG may be a useful nutraceutical as a preventive or palliative for various inflammatory diseases is extensive, it is mostly circumstantial rather than definitive, though many examples exist of the benefits of mushrooms in combating the results of oxidative stress.

Without mechanisms, finding that the concentration of a dietary metabolite X is low in disease Y does not mean that giving it might be of benefit in the prevention, delay or cure of that disease, although cases can clearly be made when X is a vitamin, or oxidative stress is known to be a damaging component of Y^(8,348)^. Thus far, we lack examples in which ERG is found both to be low in individuals with a particular syndrome and where exogenous administration effects functional improvements, although – as reviewed above – we often have one or the other.

To assess definitively any health benefits of ERG, the ‘gold standard’ of randomised controlled trials may take time and money, but – as with mushrooms^(335,563)^ – are beginning. One trial with pure ERG has been registered^(564)^.

## Note added in proof

A recent paper indicates that ERG relieves the effects seen in a rat model of the pregnancy disorder pre-eclampsia^(597)^.
